# Predictors of Professional Help-Seeking Intention Toward Depression Among Community-Dwelling Populations: A Structural Equation Modeling Analysis

**DOI:** 10.3389/fpsyt.2022.801231

**Published:** 2022-02-24

**Authors:** Xin Yi Li, Qian Liu, Pan Chen, Juan Ruan, Xuan Gong, Dan Luo, Yang Zhou, Cong Yin, Xiao Qin Wang, Lianzhong Liu, Bing Xiang Yang

**Affiliations:** ^1^School of Nursing, Wuhan University, Wuhan, China; ^2^Wuhan Mental Health Center, Wuhan, China; ^3^Department of Psychiatry, Renmin Hospital of Wuhan University, Wuhan, China; ^4^Population and Health Research Center, Wuhan University, Wuhan, China

**Keywords:** professional help-seeking intention, help-seeking attitudes, stigma, depression knowledge, structural equation modeling, depression

## Abstract

**Background:**

A low intention of professional help seeking hinders the effective treatment of depression. The factors are from the perspectives of the social, family, and individual; however, an understanding of how they interact to predict professional help-seeking intention (PHSI) is not clear.

**Objectives:**

The objectives of the study was to investigate PHSI toward depression in a Chinese community-dwelling population and construct a predictive model of the PHSI to explore the various factors involved.

**Methods:**

Stratified random sampling and Kish table methods were used to identify 2,000 community residents. Participants completed a series of questionnaires to measure general characteristics, PHSI, professional help-seeking attitude, depression stigma, depression knowledge, family function, and depression symptoms. Analyses included descriptive statistics and Pearson correlation analysis using SPSS 26.0 and a Structural Equation Model using Amos 22.0.

**Results:**

The score of the PHSI was 14.92 ± 9.574. Professional help-seeking attitude (*r* = 0.291, *p* < 0.001) and depression knowledge (*r* = 0.077, *p* = 0.002) were positively related to PHSI, while a negative correlation was found between stigma (*r* = −0.149, *p* < 0.001) and PHSI. The model of the PHSI indicated a good fit with a CMIN/DF = 2.740 and RESEA = 0.032. The total effect of the influencing factors on the PHSI was listed in the following order: professional help-seeking attitude (0.676) > stigma (−0.143) > depression knowledge (0.088) > depression symptoms (−0.009) > family function (0.005). The total effect of depression knowledge on PHSI included a direct negative effect (Beta = −0.266, *p* < 0.001) and an indirect positive effect (0.354) through professional help-seeking attitude and stigma. Also, depression knowledge was negatively associated with stigma (Beta = −0.153, *p* < 0.001). Depression symptoms were negatively associated with family function (Beta = −0.282, *p* < 0.001), depression knowledge (Beta = −0.252, *p* < 0.001), and stigma (Beta = −0.102, *p* < 0.001), indicating that people with less severe depression symptoms had good family function, depression knowledge, and higher stigma. Family function contributed a positive effect on depression knowledge directly (Beta = 0.145, *p* < 0.001) and a totally positive effect (0.033) on stigma.

**Conclusion:**

The PHSI toward depression is low among Chinese community residents. Professional help-seeking attitude, depression knowledge, and family functioning were facilitators of PHSI, and stigma and the severity of depression symptoms were barriers to PHSI. This study provides reference for the development of policies and guidelines to promote community residents to actively seek professional mental health help. Future policies can focus on multicollaboration among the government, mental health services, and families to increase the mental health resources, improve family functioning, enhance mental health literacy (MHL) of the public, and reduce depression stigma to ease the burden of this mental health issue.

## Introduction

Over 264 million people experience depression worldwide (1). In China, the lifetime prevalence of depression and major depressive disorder was 6.9 and 1.6%, respectively (2, 3). A low rate of help-seeking behaviors (4) and delayed help seeking (5) hinders timely and effective treatment for individuals with depression. Seeking help from professional resources is recognized as more effective to prevent and manage mental health problems and protect individuals against suicide risk factors (6, 7). It can promote the cognition of the event, coping skills, and more interpersonal support of the individual to solve the emotional crisis and, thus, reduce suicidal ideation (8, 9).

The theory of planned behavior (TPB) proposed that intention is a strong predictor of behavior (10). The stronger the intention of an individual to engage in a specific behavior, the more likely he is to engage in the behavior. Professional help-seeking intention (PHSI) is defined as the subjective possibility of an individual to seek help from mental health professionals (MHPs) (11). Previous studies have found a low intention to seek professional help, which corresponded to low rates of help-seeking behaviors (12–14). In America, 33.4% of respondents definitely seek professional help (15), and another study showed a lower rate that only 11% of participants expressed willingness to contact therapists (16). An 18.7% of participants with depressed mood showed strong PHSI in Japan (17). In Europe, 25% of participants showed the intention to seek help from a psychologist/psychotherapist (18). In Australia, 40% of respondents would seek help from psychologists and 34% from psychiatrists (19), and the rate was 22.8 and 24.9%, respectively, in Hong Kong, China (20). Another survey in China among pregnant women with probable depression or anxiety during COVID-19 indicated that only 19% of the sample intended to seek professional help (21).

Factors influencing PHSI appear to arise from perspectives centered on the society, family, and the individual. Socially, the accessibility and availability of mental health services (8, 22), the type of care provided by the mental health service (23), social environments, such as a neighborhood context (24), school settings (22), and health insurance policy covering mental illness (25), are common factors related to PHSI. Family factors had a limited focus in research. Parents play a role in professional help seeking for mental health problems of their children (26). The perception of family regarding help seeking, psychiatric history, and better health literacy was significantly associated with the help-seeking intention (17). Currently, family function has become a main concept in research exploring family role in diseases and the relationship between them (27). A study found that family function as a good source of social support is associated with positive professional help-seeking attitude (28). Based on the TPB, attitude is a strong predictor of intention. Given that, we propose a hypothesis that “Family function is the facilitator of PHSI directly and it may have an indirect effect on PHSI *via* help-seeking attitude” (Hypothesis 1a).

Individually, gender, age, education, economic level, characteristics of the mental illness, coping methods to solve problems, help-seeking experience, and adequate informal sources (23) have varying influences on PHSI. Among them, when referring to the severity of symptoms, the result is inconsistent (23). Some studies reported that the more severe the depression symptoms are, the greater the willingness to seek help (16), while others found the opposite: that the symptoms of hopeless and suicidal ideation in depression can decrease the motivation to deal with the problem (29) and, thus, lower the intention to seek professional help. Based on most findings, we propose the hypothesis “Depression symptoms are the barriers to PHSI” (Hypothesis 2a).

Stigma and mental health literacy (MHL) are both social and individual factors related to professional help seeking (30, 31). Stigma as a recognized help-seeking barrier (32, 33) was defined as an undesirable stereotype toward people with obvious physical or behavioral characteristics, and having tarnished their reputation (34). On this basis, Jones et al. emphasized the “mark” in the developmental process of stigma, which means if a person has shameful and unpleasant features, others will mark them as unusual, and stigma comes with it (35), including labeling, stereotyping, cognitive separation, emotional reactions, status loss, and discrimination from the perspective of social psychology (36). In most current studies, stigma was always divided into two dimensions: one is personal stigma, representing the personal attitudes of a participant toward the person described in the vignette; another one is perceived stigma, representing the beliefs of the participant about the attitudes of other people toward the person described in the vignette (37). Negative public perception has linked mental disorders with people being weak, incompetent, lacking abilities, or as failures, resulting in discrimination (38). These negative reactions discouraged people to seek help (39). Self-stigma develops when such stereotypes are applied to oneself, which can cause internalized devaluation and disempowerment (40). People with depression with stigmatizing attitudes can experience self-abasement, shame, and social withdrawal (41), which hinders their ability to seek professional help (38). In addition, there is evidence that help-seeking attitude played a mediating role between stigma and PHSI (42). So, we propose that stigma can predict PHSI directly and may have an indirect effect on PHSI *via* professional help-seeking attitude (Hypothesis 3).

MHL was defined as “*knowledge and beliefs about mental disorders which aid their recognition, management, or prevention*.” It includes the knowledge of ways of preventing mental disorders, strategies of effective self-help for minor problems, recognition of the illness development, help-seeking options, and first aid skills to assist others who are in risk of mental disorder (43). People with poorer MHL, such as lack of knowledge about mental illness and low recognition of mental health symptoms, were less likely to seek formal help for depressive symptoms (22, 44). A survey showed that 60% of the participants agreed that their delayed help seeking was caused by a lack of knowledge about mental health problems. The theory of knowledge–attitude–practice (KAP) revealed that healthy knowledge is an important basis for developing positive attitudes and further promoting behavior change (45). People with poor MHL may lead to increased stigma about mental illness (46). One model of help-seeking intention found that sociodemographic factors can directly influence health literacy and have an indirect effect on formal help-seeking intention (24). So we propose the Hypothesis 4 “Depression knowledge is a facilitator of PHSI directly, and it may have indirect effect on PHSI *via* stigma and professional help-seeking attitude.”

With regard to the family and individual factors, research is scarce, and the findings are inconsistent. A recent study showed that family function may be related to increasing stigma (37), while prior studies suggested a negative relationship (47). Thus, Hypothesis 1 is supplemented with “Family function is the facilitator of PHSI and it may have indirect effects on PHSI *via* stigma and professional help-seeking attitude.” Similarly, depression symptoms are reported that it is associated with stigma and help-seeking attitude (28, 48), so Hypothesis 2a is supplemented with “Depression symptoms are the barriers to PHSI and it may have indirect effects on PHSI *via* stigma and professional help-seeking attitude” (Hypothesis 2). These factors of the PHSI also interact with each other. Previous research has mostly concentrated on exploring the dependent effect while controlling for other confounding factors. However, how these variables work together to predict help-seeking intention is not clear. Thus, this study aims to explore the effect mechanism of these factors on PHSI toward depression and how the PHSI can be strengthened.

## Methods

### Study Design and Sample

This study is a cross-sectional survey between January and December 2017, which was conducted in Wuhan, a major city in central China. Data were obtained from community residents in 7 districts and 40 communities. Inclusion criteria were minimum age of 15 years and able to read and write in Mandarin. Participants with severe somatic illness or other psychosis and mental impairment due to substance dependence were excluded.

The sample procedure was divided into three steps to determine the target communities, households, and individuals. A stratified random sampling method was applied in the sampling process. First, 40 communities from seven central districts of Wuhan were selected using an Excel-generated random number table. Second, a systematic sampling method was used to identify 50 target households in each community. A Kish selection table method to determine target individuals from every household survey was then used in the third sampling stage. Every household was assigned a random Kish code (A, B1, B2, C, D, E1, E2, and F), corresponding to the family member code in family registration form to determine the target individual. Finally, a total of 2,000 potential participants were asked to voluntarily complete a self-assessment questionnaire. Small gifts were provided in gratitude for the participation of an individual. This study is a part of the “Cross-sectional survey of depression comprehensive prevention and management project.” More details about the sampling procedure are introduced in another study (28). The sample size should be at least five times the free parameters in SEM (49). In this study, the number of free parameters was 47, so the minimum sample size was 235. Due to this study involving many professional questionnaires and requiring household face-to-face interviewing, the investigators received professional training uniformly, including the skills of interviewing and the contents of questionnaires.

### Measurements

#### Sociodemographic Characteristics

Participants completed a researcher-designed questionnaire to collect sociodemographic information, which included age, gender, nationality, religious affiliation, employment status, occupation, education level, and marital status.

#### General Help-Seeking Questionnaire

The PHSI of the community residents for psychological problems was measured by the General Help-Seeking Questionnaire (GHSQ) (50). It was developed by Wilson and was confirmed that it provides a suitable method for measuring help-seeking intentions and supports the specification of different problem types and different help sources (e.g., partners, parents, friends, and MHPs). It has been applied to a Chinese community with a Cronbach's α of 0.91 and test–retest reliability of 0.95 (51). In the current study, five items of the GHSQ were used to assess the intentions of the Chinese community residents to seek help from MHPs to cope with emotional problems, which showed good reliability with a Cronbach's α of 0.954. Participants were asked the following questions: If you had a negative emotional problem, such as depression or anxiety, how likely would you seek help from the following list of resources? The professional resources include psychiatrist at a general hospital, psychiatrist at a psychiatric hospital, psychologist at a general hospital, psychologist at a psychiatric hospital, and psychologist in a school or other institution. These items used a seven-point Likert scale with a score ranging from 1 (extremely unlikely) to 7 (extremely likely). The total score of the five items ranges from 5 to 35 with the higher scores indicating greater PHSI.

#### Attitudes Toward Seeking Professional Psychological Help Scale—Short Form

The Attitudes Toward Seeking Professional Psychological Help Scale—Short Form (ATSPPH-SF) was developed by Fischer and Farina (52). The scale was used to measure the ATSPPH of the participants, and it is a four-point Likert scale. The total score of the scale ranges from zero to 30 with higher scores indicating a better help-seeking attitude. The Chinese version of the ATSPPH-SF was suggested to be valid and reliable with a Cronbach's α of 0.681, and the test–retest reliability was 0.895 (53).

#### Depression-Specific Self-Management Scale

This scale was used to measure depression self-management of participants (54). It consists of nine items with two subscales: the first four items evaluate the specific knowledge of depression of the respondents, and the following five items evaluate the specific activities of the respondents for improving depressive symptoms. Each subscale uses a five-point Likert scale. The current study selected the subscale of Depression-Specific Self-Management (DSSM)—Knowledge, which covers the following themes: the treatment of depression, the knowledge of antidepressants, the literacy of help seeking, and the recognition of depression symptoms. The total score of depression knowledge ranges from 4 to 20 points with a higher score indicating a higher level of depression knowledge. The Cronbach's α was 0.578.

#### Depression Stigma Scale

The Depression Stigma Scale (DSS) was used to access the stigma related to depression in participants (55). The original English version of the DSS was authorized for translation into Chinese, which has a good reliability and validity (37). It has a five-point Likert scale, which ranges from 0 (strongly disagree) to 4 (strongly agree). The total scores were aggregated for all items, with higher scores representing higher levels of stigma. The Cronbach's α is 0.809 in the current study.

#### Center for Epidemiological Studies Depression Scale

The Center for Epidemiological Studies Depression Scale (CES-D) is a 20-item instrument used to screen depression during the past week (56). In the current study, it was used to assess the severity of depressive symptoms. Each item uses a four-point scale ranging from 0 (no or hardly) to 3 (almost always). The total scores range from 0 to 60 with higher scores indicating greater depressive symptoms. The instrument attained excellent reliability both in the English and Chinese versions, with Cronbach's α of 0.91 (57) and 0.90 (58), respectively.

#### Family APGAR Index

To measure the satisfaction of an individual with family functioning, the current study used the Family APGAR index developed by Smilkstein (59). It consists of five items standing for Adaptation, Partnership, Growth, Affection, and Resolution, respectively. It uses a three-point Likert scoring from 0 (hardly ever) to 2 (almost always), and the total score ranges from 0 to 10 with higher scores indicating better family function. The Chinese version of the Family APGAR index has been applied widely in China, and the Cronbach's α was 0.94 (60).

### Statistical Analysis

Data analysis was conducted using SPSS 26.0 and Amos 22.0 (61). Frequency and percentage were used to describe the categorical variables; means and standard deviations were used to describe the continuous variables. Pearson correlation analysis was used to explore the relationship among variables. Amos 22.0 was used to establish the SEM. Test for multivariate normal distribution was required; skewness and kurtosis values within the range of from −2 to 2 indicate that variables were normally distributed. A maximum likelihood (ML) method to estimate parameters when the data meets normal distribution was used (62). The model was modified and optimized according to the modified index. The goodness-of-fit of the model was evaluated synthetically by various fitting indexes. A good-fitting model requires that the value of CMIN/*df* is <3, and IFI (incremental fit index), GFI (goodness of fit index), and CFI (comparative fit index) are >0.90, and the value of RMSEA (root mean square error of approximation) should be under 0.05. Results were considered statistically significant with a level of *p* < 0.05. Questionnaires with four or more missing values related to the variables were excluded and considered invalid, while for a questionnaire that had less than or equal to four missing item responses, a mean completer method was used to deal with the missing values.

### Ethical Principles

This study was approved by the Wuhan University School of Medicine Institutional Review Board. Based on the principle of voluntary participation and withdrawal from the study at any time, subjects signed informed consents prior to participation.

## Results

### Sociodemographic Characteristics

A total of 2,000 questionnaires were distributed; 1,732 questionnaires were returned (response rate: 86.6%) and 1,656 questionnaires were valid (effective callback rate: 95.6%). The general characteristics are shown in Table 1. The average age of participants was 30.27 ± 15.541 years, ranging from 16 to 89 years.

**Table 1:** Sociodemographic characteristics of the sample (*N* = 1,656).

**Characteristics**	***n***	***%***
**Gender (*****n*** **=** **1,656)**		
Male	543	32.8
Female	1,113	67.2
**Nationality (*****n*** **=** **1,655)**		
Han	1,535	92.7
Other	120	7.3
**Religious affiliation (*****n*** **=** **1,653)**		
No	1,581	95.6
Yes	72	4.4
**Education level (*****n*** **=** **1,656)**		
Less than high school	168	10.1
Junior high school/high school/some college	409	24.7
Bachelor's degree or higher	1,079	65.2
**Employment status (*****n*** **=** **1,656)**		
Unemployed/laid-off/retired	285	17.2
Full time/part time	1,371	82.8
**Occupation (*****n*** **=** **1,604)**		
Skilled worker/farmer/business man/other	245	15.3
General company/state-owned enterprise or public institution staff/civil servant	442	27.5
Student	917	57.2
**Marital status (*****n*** **=1,651)**		
Single/separated/divorced/widowed	1,079	65.4
Cohabiting/married/remarried	572	34.6

### Professional Help-Seeking Intention

The score of the PHSI scale is shown in Table 2. The total score was 14.92 ± 9.574, which ranged from 5 to 35. Item 3 “Psychologists from general hospital” had the highest score (3.02 ± 2.081), while item 5 “psychologists from school or other institutions” had the lowest score (2.93 ± 2.078). For all the five items of the scale, over 60% of the respondents had scores ≤ 3, indicating that they were unlikely to seek such professional help.

**Table 2:** Score of professional help-seeking intention (PHSI).

**Items**	***M***	***SD***	**≤3**	**>3**
			***n* (%)**	***n* (%)**
1. Psychiatrists from general hospital	2.97	2.069	1,028 (62.1)	628 (37.9)
2. Psychiatrists from psychiatric hospital	2.98	2.094	1,039 (62.7)	617 (37.3)
3. Psychologists from general hospital	3.02	2.081	1,006 (60.7)	650 (39.3)
4. Psychologists from psychiatric hospital	3.01	2.091	1,011 (61.1)	645 (38.9)
5. Psychologists from school or other institutions	2.93	2.078	1,038 (62.7)	618 (37.3)

*PHSI, professional help-seeking intention; M, mean; SD, standard deviation*.

### Pearson Correlation Analysis

Pearson correlation analysis showed that professional help-seeking attitude (*r* = 0.291, *p* < 0.001) and depression knowledge (*r* = 0.077, *p* = 0.02) were positively related to the PHSI, while a negative correlation was found between stigma (*r* = −0.149, *p* < 0.001) and PHSI. There were no correlation relationships between family function, depression symptoms, and PHSI (see Table 3).

**Table 3:** Results of correlation analysis.

**Variables**	**Help-seeking attitude**	**Stigma**	**Depression knowledge**	**Family function**	**Depression symptoms**
Help-seeking attitude	1				
Stigma	−0.191^**^	1			
Depression knowledge	0.217^**^	−0.084^*^	1		
Family function	0.078^*^	0.042	0.123^**^	1	
Depression symptoms	−0.092^**^	−0.073^*^	−0.212^**^	−0.265^**^	1
PHSI	0.291^**^	−0.149^**^	0.077^*^	0.023	0.010

** *Correlation is significant at the 0.01 level (two tailed)*.

* *Correlation is significant at the 0.05 level (two tailed)*.

### The Structural Equation Model of Professional Help-Seeking Intention

**Figure 1:**
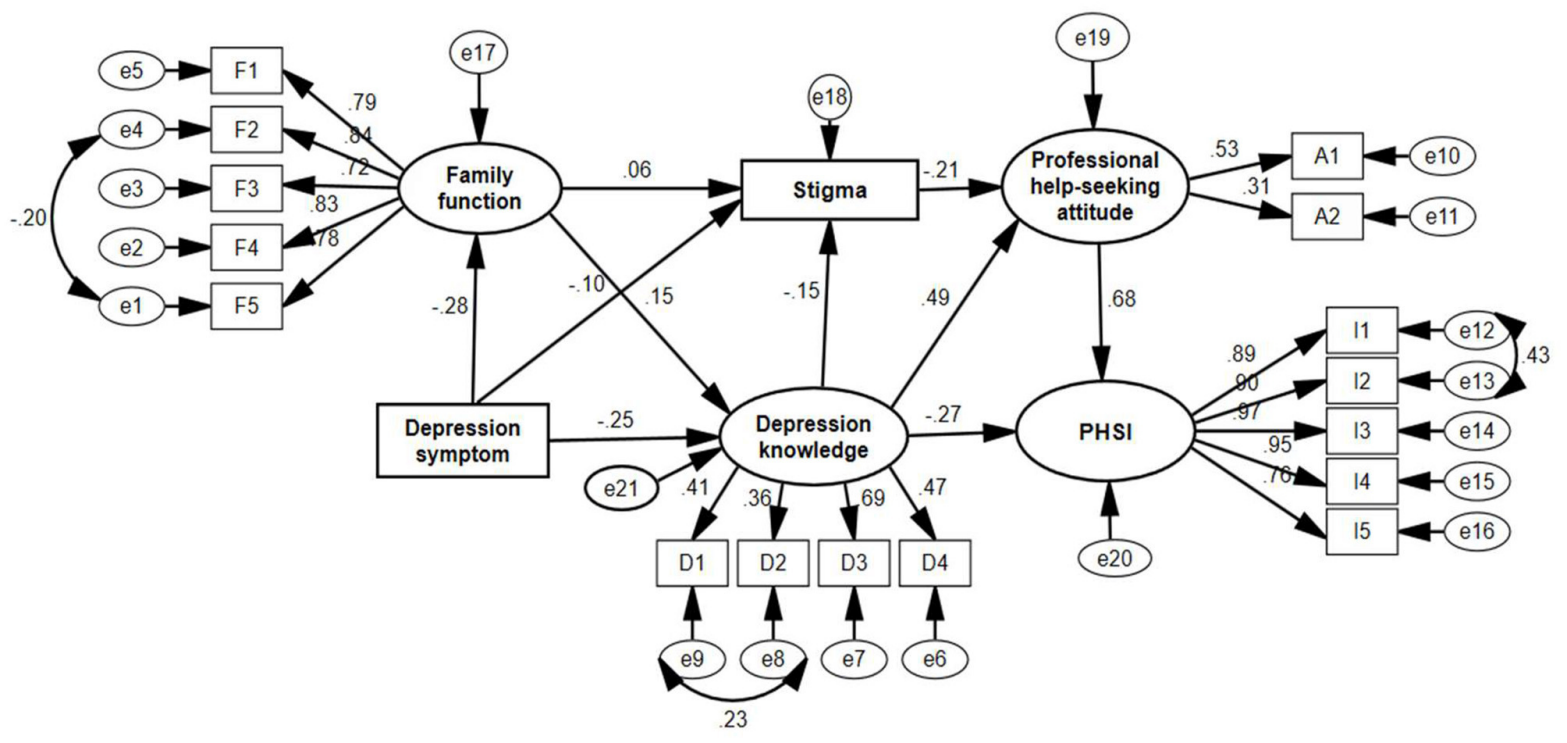
The structural equation model of professional help-seeking intention (PHSI).

The final model is shown in Figure 1. The test for multivariate normal distribution showed that the kurtosis coefficients of all variables ranged from −1.340 to 0.575, the skewness coefficients ranged from −1.036 to 2.022, indicating that the data satisfied the assumption of multivariate normality. The final model was an over-identified model (*df* = 124 > 0, chi-square = 339.752, *p* < 0.001) according to the *t*-rule [59], and there are no outliers >1 in the model, and it had a good fitness with a CMIN/*df* = 2.740, RMSEA = 0.032, GFI = 0.977, AGFI = 0.968, PGFI = 0.709, NFI = 0.978, RFI = 0.973, IFI = 0.986, and CFI = 0.986.

The effect of the influencing factors on the PHSI is shown in Table 4, which included the total, direct, and indirect effects. The total effect of the influencing factors on the PHSI was listed in the following order: professional help-seeking attitude (0.676) > stigma (−0.143) > depression knowledge (0.088) > depression symptoms (−0.009) > family function (0.005). Professional help-seeking attitude was the most important factor to predict PHSI (Beta = 0.676, *p* < 0.001).

**Table 4:** Effects among other variables (Beta, *p* < 0.05).

	**Effect**	**PHSI**	**Professional help-seeking attitude**	**Stigma**	**Depression knowledge**	**Family function**
Professional help-seeking attitude	Total	0.676				
	Direct	0.676				
	Indirect	0.000				
Stigma	Total	−0.143	−0.211			
	Direct	0.000	−0.211			
	Indirect	−0.143	0.000			
Depression knowledge	Total	0.088	0.524	−0.153		
	Direct	−0.266	0.491	−0.153		
	Indirect	0.354	0.032	0.000		
Family function	Total	0.005	0.064	0.033	0.145	
	Direct	0.000	0.000	0.055	0.145	
	Indirect	0.005	0.064	−0.022	0.000	
Depression symptoms	Total	−0.009	−0.129	−0.073	−0.293	−0.282
	Direct	0.000	0.000	−0.102	−0.252	−0.282
	Indirect	−0.009	−0.129	0.029	−0.041	0.000

Family function contributed the least effect (0.005) on PHSI among all the variables, and the effect was indirect. It also contributed a positive effect on professional help-seeking attitude indirectly (0.064) and on depression knowledge directly (Beta = 0.145, *p* < 0.001). Family function had a totally positive effect (0.033) on stigma, including a positive direct effect (Beta = 0.055, *p* = 0.043) and a negative indirect effect (−0.022). So, the Hypothesis 1 was partially supported. As the result showed, depression symptoms had a totally negative effect (−0.009) on PHSI indirectly, and the effect of depression symptoms on all other variables was negative, which partially supported Hypothesis 2. Depression symptoms were negatively associated with family function (Beta = −0.282, *p* < 0.001), depression knowledge (Beta = −0.252, *p* < 0.001), and stigma (Beta = −0.102, *p* < 0.001), indicating that people with less severe depression symptom had good family function, depression knowledge, and higher stigma.

Stigma had a negative indirect effect (−0.143) on PHSI *via* negatively related to professional help-seeking attitude (Beta = −0.211, *p* < 0.001), while there was no direct relationship between stigma and PHSI, which partially supported Hypothesis 3. The total effect of depression knowledge on PHSI was positive (0.088), including a direct negative effect (Beta = −0.266, *p* < 0.001) and an indirect positive effect (0.354) through professional help-seeking attitude and stigma. So, Hypothesis 4 was supported. Also, depression knowledge was negatively associated with stigma (Beta = −0.153, *p* < 0.001).

## Discussion

The PHSI of depression is not positive in a Chinese community-dwelling population. To the knowledge of the researchers, this study constructed a new predictive model of PHSI. Based on the theories of TPB and KAP, this study added the variables of professional help-seeking attitude, stigma, and depression knowledge, and additionally, it also added the key factors of family function and severity of depression symptoms in the model. This model has a good fitness and adequately illustrates the interrelationship among the factors.

### The low Intention of Professional Help-Seeking

This study showed that for every type of MHP mentioned in this study, over 60% of respondents indicated that they were unlikely to seek such assistance. The similar low help-seeking situation was also reported in other psychiatric disorders, such as anxiety. Over 40% of participants never considered seeking help for their anxiety problems in the German community residents (63). While in the schizophrenia population, the rate was higher, 63.8% of them sought medical help in Ethiopia (64) and 64.6% in China (65). This difference may be related to the perceived attribution and characteristics of different psychiatric disorders (64). The inactive PHSI can be explained by various reasons. First, the accessibility and availability of mental health services is limited (6). The Comprehensive Mental Health Action Plan 2013–2030 proposed by WHO points to this serious problem, especially in low- and middle-income countries (66). In China, there is a serious deficit of mental health resources (67) including manpower and services. There were 2.02 psychiatrists and 4.19 psychiatric nurses per 100,000 population in 2015 compared with high-income countries, in which the ratio was 13.06 and 23.49 (67, 68). Moreover, mental health services in China are mainly concentrated in psychiatric departments of general hospitals (43.19%) and psychiatric specialty hospitals (42.06%), and the proportion of primary-level medical and health institutions is only 9.98% (69), which leads to a relative shortage of community mental health resources. The Comprehensive Mental Health Action Plan 2013–2030 also emphasized the necessity of providing comprehensive and integrated community-based mental health and social care services (66). Second, cultural attribution has limited the expression and recognition of mental health problems. Chinese are deeply influenced by Confucianism, which attaches importance to the concepts of harmony (70), collectivism (71), and the acceptance of destiny (72). Also, the theory of “yin” and “yang” from Taoism emphasizes balance and is historically used to explain the various phenomena of human physiology and pathology, and animism, which explains that everything in the world possesses souls and spirits (73). Thus, they often try traditional treatments, such as herbs and acupuncture first, or find ways to dispel evil spirits (74). Finally, the severity and complexity of the personal psychological problems (14), the negative perception such as being skeptical about the effectiveness of psychological help or the competence of MHPs (75), and previous unpleasant help-seeking experience (76) are also reasons for not seeking professional help.

### Effects of Help-Seeking Attitude, Stigma, Family Function, and Depression Symptoms on Professional Help-Seeking Intention

Professional help-seeking attitude contributed the greatest effect on PHSI (10). In addition, it also played a mediator role in the relationship between other variables and PHSI. Consistently, stigma can negatively influence PHSI in depression by help-seeking attitude (77). It was also a help-seeking barrier in anxiety disorder (78, 79), posttraumatic stress disorder, social phobia, or psychosis (80). Previous studies suggested that perceived stigma was higher than personal stigma for depression in China (37), which was consistent with that of Germany (81). On one hand, increasing stigma can hinder the early recognition of depression, lower the self-identification of having depression, and decrease perceived need for professional help (82, 83). On the other hand, a sense of shame, embarrassment, and fear of discrimination are the primary intuitive feelings (38). There is evidence that about 44% of the respondents felt that they would be embarrassed to see a psychiatrist for their depression problems in Australia (84). Mental illness is often labeled as “sick, neurotic, incompetent, weak, punishment, or ‘*dian kuang*' (in Chinese)” (19, 85). Maintaining “face” and harmony are the most important considerations in a Chinese collectivism society (77), which leads to the individual preferring to conceal their emotional problem rather than seek help. Thus, it is necessary to take effective measures to reduce the depression stigma among the general community-dwelling population and then strengthen their PHSI when they experience an emotion problem (86). As research has pointed out, improving overall MHL is a common approach to improve stigmatizing attitudes (84, 87). Antistigma campaigns, such as the Nuremberg Alliance Against Depression in Germany and “Beyondblue” in Australia, a national depression initiative, aim to promote the recognition and provide information on mental health issues, and other types of interventions have also made great improvements, such as the Internet-based cognitive behavior therapy, which has shown a significant decrease in personal stigma (88), especially in adolescents who are frequent users of the Internet (84). Thus, future interventions to reduce stigma could promote psychotherapy along with enhanced MHL, and the approach can include online and offline services according to the target population.

Family function and depression symptoms can influence PHSI indirectly by the mediating effect of depression knowledge, stigma, and attitude. Better family function is related to greater depression knowledge, and families are always the first source of help seeking when someone suffers from emotional distress (89). Family members can offer proper advice, share the experience and information about mental health, come up with guidelines on problem solving, and participate in decision making (90). This study also showed that the more severe the depression symptoms are, the poorer the intentions and attitudes to seek professional help (79). The complex mechanism of action includes exerting negative effect on family function, stigma, depression knowledge, and professional help-seeking attitude.

### Effect of Depression Knowledge on Professional Help-Seeking Intention

The recognition of symptoms is the first step to seek help from professional sources (91). A study in Vietnam showed that among those who correctly identified depression, 82.1% would seek help (92). In this study, it is worth noting that depression knowledge is negatively associated with PHSI in the direct path. This can be explained by the fact that the model of this study contained more variables that mutually affect PHSI, so the role of depression knowledge on PHSI is influenced by other variables. Another explanation may be that people who have a good command of mental health knowledge need not seek professional help; they can manage their mood well on their own or seek some informal help such as from families or friends (92). There was also a study that elicited that knowledge is not a predictor of PHSI, and the explanation given by the researcher for this disparity might be that the methods to measure depression knowledge were different (79).

In the indirect paths, greater depression knowledge can positively affect PHSI through exerting a negative effect on stigma (46) and a positive effect on professional help-seeking attitude (28). This relationship was consistent with a similar model of help-seeking intention (77). The facilitation effect was stronger than the hindrance effect. Thus, the total effect of depression knowledge on PHSI is positive (93). People with lower depression knowledge may be unable to identify the symptoms of depression (91) and not know where to go to seek professional help (94); thus, the motivation of help seeking would be decreased. Generally, the literature on MHL of different cultural and national groups has shown a similar low recognition of various disorders (46, 95). Most people were unable to figure out the specific mental disorder or had difficulty understanding professional terms (96, 97). Some people normalized mental illness and believed it can go away on their own (98). A survey reported that 43.8% of the participants believed that a lack of self-discipline and willpower is one of the main causes of mental illness, and 21.9% did not consider depression as a mental illness (99). One comparative study showed that 14% of the participants who were Chinese-speaking Australians could correctly identify major depression symptoms, which was lower than Japanese participants (22.6%) and Australian samples (67.6%) (97). Consistent with one study in Singapore (100), this study reemphasized the finding that improving overall MHL is a common approach to improve stigmatizing attitudes (87), that is, greater knowledge may diminish the stigma related to depression, and then people are more willing to seek professional help (101, 102).

### Direct Positive Effect of Family Function on Stigma

This is an interesting finding that people with good family function had a higher level of stigma. This is contrary to most previous studies (47, 103). It could be explained by the Chinese culture. The Chinese family emphasizes cohesion in contrast with the independence belief in Western culture (104). People with good family function have strong family cohesion and kinship ties, and they are more likely to be influenced by family values (105). The more support a person receives from family, the more willing they are to maintain a harmonious and close relationship by obeying the thoughts of families (106). Thus, individuals with good family function are more likely to believe the family views, though their family has a misunderstanding of mental illness. There is evidence that the culture of family collectivism was associated with more discrimination and prejudice against people with depression (104). Traditional Chinese people place importance on the family as a unit. The collective honor of the family is more important than individual feelings. If someone in the family suffers from mental illness, the family would be considered problematic, and this can even rise to the level of a moral issue (36). For instance, parents whose children have mental illness may be considered as bad parents, even being considered the result of a bad bloodline (107). A history of family mental illness may damage the reputation of the entire family (73, 108), and family members also may experience “affiliate stigma” because of a close family member having mental illness (108). Family members would be unwilling to recommend their family members with mental illness to seek treatment because of the sense of family shame (107). Stigma of individuals may be increased due to guilt and the fear of disgracing the family honor and their ancestors (109), which may lead to their concealing their problems for the sake of keeping the face of the families.

Comprehensively, although good family functioning enhances stigma, to some extent, increasing depression knowledge can weaken the positive effects of family functioning on stigma, and the effect of family functioning is stronger for depression knowledge than for stigma. Therefore, family functioning was positively associated with attitude toward seeking professional help. Under the combined influence of stigma, depression knowledge, and professional help-seeking attitude, there is a positive relationship between family functioning and PHSI. Thus, the family factor should be taken into consideration in developing interventions for increasing depression knowledge and reducing stigma to promote professional help-seeking attitude and intention.

### Effect of Depression Symptoms on Stigma

A total negative relationship was shown in this study between depression symptoms and stigma. This is consistent with previous research that indicated that people who experienced depression had a lower personal stigma (48, 110). The possible reason is that people who have more severe symptoms are more apt to attribute psychiatric symptoms as somatic diseases (111). Thus, the stigma related to mental disorder is avoided. Other research revealed that people with depression are more likely to compare themselves with similar groups, thus, it can protect people against stigma since people with similar features exhibit functioning in the same way and will maintain their self-esteem (40). Intact self-esteem and high group identification can promote positive self-perception; thus, people who have depression and experience stigma may identify with peers and defend themselves from the harmful effects of stigma (40, 112).

In addition, family function mediated the relationship between the depression symptoms and stigma, and, thus, reinforces the stigma to some extent. There was a negative relationship between the severity of depression symptoms and family function, which is supported by previous studies (113). One study in mainland China showed that the family function of one family with a depressed member was poorer than a normal family, and depressed individuals had less satisfaction about their family (114). It is a warning that future interventions should focus more on families whose family members have severe depressive symptoms and improve their family functioning. More mental health knowledge education should be spread to strengthen public identification with depression and, thus, to reduce the stigma. Future antistigma interventions could contain online and offline approaches according to the target population, thereby promoting greater intention to seek professional help.

### Strengths and Limitations

To the best of our knowledge, this study is the first epidemiological survey of help-seeking intentions toward MHPs in a general community-dwelling population in China and the first study to construct an SEM of PHSI and explore the mechanism among influencing factors. It was conducted based on a large-scale population survey, which had a large sample size. The results can provide valuable basic data for mental health prevention and treatment, and offer the theoretical reference to make target interventions to promote improved mental health. Existing PHSI-related research remains scarce in China, and this study enriches the research in this field to some extent.

There are several limitations of this research. First, this is a cross-sectional survey, a causal relationship between help-seeking intention, and its influencing factors cannot be drawn. Second, there may be bias since participants may respond in a socially desirable manner when using self-rating scales including depressive symptoms over the past week, personal and public stigma related to depression, and help-seeking attitude and intention toward professionals. Third, this study was completed in one city in central China, which may limit the generalization of the findings to other areas of the country. Finally, the influence factors of PHSI are various; this model explores a part of these factors, so future studies could take more variables into consideration to get a better model of PHSI, such as culture and personality factors.

## Conclusion

Professional help-seeking intention toward depression is low among Chinese community residents. Professional help-seeking attitudes, depression knowledge, and family functioning were facilitators of PHSI, and stigma and the severity of depression symptoms were barriers to PHSI. This study provides a reference for the development of appropriate policies and guidelines in the future to promote community residents to actively seek professional help for mental health. Efforts should be made under multicollaboration according to the Mental Health Plan 2013–2030 (66). For the government, they can increase the mental health resources, provide more mental health information and antistigma campaigns based on culture, and protect the rights and opportunities of individuals with mental disorders. For mental health services, they can identify early and intervene emotional or behavioral problems by a timely screening program, especially in childhood and adolescence, and strengthen the training of MHPs to promote greater trust between those who seek services and the providers. For families, improve the family function to create healthy living conditions and social support.

## Data Availability Statement

The raw data supporting the conclusions of this article will be made available by the authors, without undue reservation.

## Ethics Statement

The studies involving human participants were reviewed and approved by Wuhan University School of Medicine Institutional Review Board. Written informed consent to participate in this study was provided by the participants' legal guardian/next of kin.

## Author Contributions

BXY, XQW, LL, XYL, PC, QL, and JR designed the study and wrote the research protocol. XYL, PC, XQW, QL, BXY, LL, JR, XG, DL, YZ, and CY did the literature review, managed the field survey, quality control, and statistical analysis and prepared the manuscript draft. PC, XQW, BXY, and LL contributed to the revisions in depth for the manuscript. XG, DL, YZ, CY, and BXY supervised the survey and checked the data. All authors contributed to and approved the final manuscript.

## Funding

We appreciate the grant support from the National Natural Science Foundation of China (No. 72174152), the Wuhan Health and Family Planning Commission Research Fund (WX17B16), Hubei Key Laboratory Opening Project of Renmin Hospital of Wuhan University (2021KFH012), Wuhan University School of Sciences Independent Research Fund (ZZKY005), the National Natural Science Foundation of China (No. 71503192), and the Young Top-notch Talent Cultivation Program of Hubei Province.

## Conflict of Interest

The authors declare that the research was conducted in the absence of any commercial or financial relationships that could be construed as a potential conflict of interest.

## Publisher's Note

All claims expressed in this article are solely those of the authors and do not necessarily represent those of their affiliated organizations, or those of the publisher, the editors and the reviewers. Any product that may be evaluated in this article, or claim that may be made by its manufacturer, is not guaranteed or endorsed by the publisher.
